# Transcription Factor Ets1 Regulates Expression of Thioredoxin-Interacting Protein and Inhibits Insulin Secretion in Pancreatic β-Cells

**DOI:** 10.1371/journal.pone.0099049

**Published:** 2014-06-04

**Authors:** Yan Luo, Fengli He, Li Hu, Luo Hai, Meifeng Huang, Zhipeng Xu, Jingjing Zhang, Zhiguang Zhou, Feng Liu, Yan-Shan Dai

**Affiliations:** 1 Metabolic Syndrome Research Center, Key Laboratory of Diabetes Immunology, Ministry of Education, the Second Xiangya Hospital, Central South University, Changsha, Hunan, China; 2 Department of Pharmacology, University of Texas Health Science Center at San Antonio, San Antonio, Texas, United States of America; University of Cincinnati, College of Medicine, United States of Ameirica

## Abstract

Long-term activation of extracellular-regulated kinase (ERK1/2) pathway has been shown to cause glucotoxicity and inhibit insulin gene expression in β-cells. Transcription factor Ets1 is activated by ERK1/2-mediated phosphorylation at the Thr38 residue. We hypothesize that Ets1 plays an important role in mediating ERK1/2 induced glucotoxicity in β-cells. We determined the role of Ets1 in Min6 cells and isolated mouse islets using overexpression and siRNA mediated knockdown of Ets1. The results show that Ets1 was localized in insulin-staining positive cells but not in glucagon-staining positive cells. Overexpression of Ets1 reduced glucose-stimulated insulin secretion in primary mouse islets. Overexpression of Ets1 in Min6 β-cells and mouse islets increased expression of thioredoxin-interacting protein (TXNIP). Conversely, knockdown of Ets1 by siRNA reduced expression of TXNIP in Min6 cells. Ets1 was associated with the txnip promoter in min6 cells and transfection of 293 cells with Ets1 and p300 synergistically increased txnip promoter reporter activity. Moreover, overexpression of Ets1 inhibited Min6 cell proliferation. Our results suggest that Ets1, by promoting TXNIP expression, negatively regulates β-cell function. Thus, over-activation of Ets1 may contribute to diet-induced β-cell dysfunction.

## Introduction

It is known that both impaired β-cell function and decreased β-cell mass contribute to the insulin secretion deficiency in patients with type 2 diabetes. Glucotoxicity plays a major role in pancreatic β-cell apoptosis, diabetic complications and progression of diabetes. The proposed mechanisms of β-cell glucotoxicity include β-cell overstimulation, oxidative stress, ER stress, protein glycation and AGE-receptor pathway, activation of the hexosamine pathway, PKC activation, inflammation, islet amyloid deposition, and hypoxia [1], [2]. Post-translational loss of MafA protein also contributes to the mechanism of glucotoxicity [3].

Activation of the ERK1/2 pathway has been shown to cause glucotoxicity [2]. ERK1/2 is required for stimulation of insulin gene expression under the normal physiological range of glucose concentrations, whereas chronic hyperglycemia for more than 24 h inhibits insulin gene transcription in an ERK1/2-dependent manner [4], [5]. Prolonged exposure of β-cells to high glucose or glucosamine induces ER stress. Following ER stress, ERK is activated through inositol-requiring 1 (IRE1)-dependent mechanisms. Glucotoxic ER stress dedifferentiates β-cells, in the absence of apoptosis, through a transcriptional response. These effects are mediated by the activation of ERK1/2 [6]. Pentose phosphate pathway metabolites also contribute to decreases in insulin gene expression and glucose-stimulated insulin secretion, and these effects depend on the activation of ERK1/2. Inhibition of ERK1/2 during chronic glucose exposure reduces accumulation of pentose phosphate pathway metabolites and partially restores β-cell function in the rat β-cell line INS-1E and human islets [7]. It has been shown that palmitate enhances glucose-induced phosphorylation of ERK1/2 and that pharmacological inhibition of ERK1/2 partially restores insulin gene expression in insulin-secreting cells and isolated islets exposed to palmitate or ceramide [8].

Recent studies have identified TXNIP (also known as TBP-2) as a mediator of oxidative stress induced β-cell glucotoxicity [9]–[12]. Oxidative stress occurs mainly due to excessive accumulation of cellular reactive oxygen species (ROS) or deficiency of antioxidant defense system. TXNIP is implicated in induction of oxidative stress through its interaction with thioredoxin, a critical redox protein in cells. Therefore, TXNIP is a key transducer of glucotoxicity, oxidative stress, and ER stress in islets [13]–[15]. High glucose also activates TXNIP expression through CHREBP transcription factor [16]. ChREBP mediates glucotoxicity by upregulating downstream target genes Fasn and TXNIP [17]. Studies using TXNIP-deficient mouse model demonstrate that TXNIP induction plays an important role in glucotoxicity and β-cell apoptosis [18], [19]. Disruption of TXNIP in obese mice (ob/ob) dramatically improve hyperglycemia and glucose intolerance. TXNIP-deficient ob/ob mice exhibit enhanced insulin sensitivity and glucose-stimulated insulin secretion (GSIS) in islets. Recent studies show that TXNIP links ER stress to NLRP3 inflammasome in β -cells [12]–[14]. TXNIP is induced by ER stress through the PERK and IRE1 pathways. TXNIP activates IL-1β production through the NLRP3 inflammasome, and mediates ER stress-mediated β cell death [12], [14].

Transcription factor Ets1 encodes E26 transformation-specific sequence and plays an important role in mediating inflammation and remodeling. Ets1 has been well studied in the regulation of different aspects of cancer cell behavior, including extracellular matrix remodeling, invasion and angiogenesis [20]. Ets1 is activated by ERK-mediated phosphorylation at T38 [21], which leads to increased affinity of Ets1 with co-activator P300/CBP and enhanced transcriptional activity of Ets1 [22], [23]. Ets1 interacts with NFAT transcription factors and facilitates nuclear entry of NFAT proteins and their recruitment to the IL-2 promoter [24]. Ets1 is involved in the regulation of TXNIP transcription induced by a synthetic retinoid CD437 in human osteosarcoma cells [25]. cAMP-PKA signaling pathway upregulates expression of Ets1, which in turn directly activates the expression of caspase-1, the enzyme that activates IL-1β by cleaving pro-IL-1β, suggesting a link between Ets1 and NLRP3 inflammasome [26].

It is unknown whether Ets1 is expressed in islets and plays a role in β-cell function. Our results show that Ets1 induced the expression of TXNIP and inhibited insulin secretion in β-cells. This work was presented to American Diabetes Association 73^rd^ Scientific Sessions in 2013 [27].

## Materials and Methods

### Materials

C57BL/6 mice were purchased from Model Animal Research Center of Nanjing University and were maintained in the accredited pathogen-free Second Xiangya hospital mice facility on a 12 h light/dark cycle. All experiments were performed following approval of the protocol by the Animal Care Research Committee of Second Xiangya Hospital.

Mouse Ets1 cDNA clone was purchased from Open Biosystems and subcloned into pShuttle vector (Clontech). Adenovirus expressing Ets1 was constructed using Adeno-X expression system (Clontech). TXNIP promoter luciferase reporter containing 1 kb upstream transcription start site was purchased from Addgene (plasmid #18759), which was deposited by Dr. Clark Distelhorst. P300 expression vector was described before [28]. Mouse Ets1 siRNA 2, 5, 6, 7 were purchased from Qiagen. Ets1 rabbit antibody (sc-111) was from Santa Cruz. Ets1-phospho T38 antibody was from Abcam. Insulin and glucagon monoclonal antibodies were from Sigma. Mouse monoclonal Anti-TXNIP was from MBL, Japan. GAPDH, Horseradish peroxidase labeled donkey anti rabbit or donkey anti mouse antibodies were from Cell Signaling (Beverly, MA).

### Methods

#### Cell Culture

Min6 cells were originally purchased from ATCC and were cultured in DMEM containing 15% FBS, 25 mM Glucose and 50 µM β-mercaptoethanol.

Min6 cells were seeded in a six-well plate and allowed to attach overnight. The following day, cells were treated with 20 µM PD98059 (Sigma). Min6 cells were incubated for 24 h before collection and analysis.

#### Islet isolation

Islets were isolated from 8 to 12 weeks old C57BL/6 male mice as described before by our laboratory [29]. Briefly, mouse islets were isolated using perfusion and digestion of pancreas with collagenase V (from Roche), density gradient purification with histoplaque (Sigma), and then hand-picked. Isolated islets were cultured overnight in RPMI 1640 containing 10% FBS, 11 mM glucose, and then switched to RPMI medium containing 3.3 mM glucose for 1 hr before treatment with high concentration of glucose. Insulin levels in the culture media were measured with an ELISA kit from ALPCO. For Ets1 induction of TXNIP studies, 200 isolated mouse islets were also cultured 16 hrs in RPMI 1640 containing 10% FBS and 11 mM glucose and switched to RPMI 1640 with 10% FBS and 20 mM glucose. Ad-EGFP or Ad-Ets1 was then added to islets in culture for 24 hrs at MOI of 100. The islets were collected for Western blot analysis of TXNIP and Ets1.

#### Western blots

Western blots were performed using equal amounts of whole cell extract protein. Briefly, cell lysates were run on SDS PAGE, proteins transferred to a nitrocellulose membrane. The membranes were incubated with the primary antibodies anti- TXNIP, anti-phospho-Ets1and anti-GAPDH, respectively, followed by Horseradish peroxidase labeled donkey anti rabbit or donkey anti mouse antibodies. Protein signal was visualized by using Immun-Star chemiluminescent kit (Bio-Rad) and quantified by Bio-Rad Imager.

#### Immunofluorescence

Tissue sections were antigen retrieved and blocked with 10% goat serum and 0.1% Triton-X 100. For Ets1, insulin and glucagon staining, slides were incubated overnight with rabbit anti-Ets1 antibody (sc-111, lot# L1708), together with mouse anti-mouse insulin monoclonal antibody (Sigma) or with mouse anti-glucagon monoclonal antibody (Sigma), followed by detection with an AlexaFluor488-conjugated goat anti-rabbit secondary antibody and AlexaFluor568-conjugated goat anti-mouse secondary antibody (Invitrogen). Sections were counterstained with DAPI. Images were captured and analyzed using the Zeiss Confocal Microscope.

#### Adenoviral infection

Adenovirus was amplified in 293 HEK cells. The adenovirus in 293 cells was collected and subjected to three cycles of freeze-thaw. Adenovirus titer was determined by using Adeno-X rapid titer kit from Clontech. Min6 cells or isolated islets were infected with Ad-Ets1 or Ad-EGFP at 100 MOI for 16 hours. The next day, the medium was changed and the cells were cultured for total 48 hours.

#### siRNA transfection

Min6 cells were transfected with Ets1 siRNA or control scramble siRNA using Metafectene Pro, a cationic liposome based transfection reagent (Biontex laboratories, Germany), which possessed high transfection efficiency in Min6 cells (not shown).

#### RT-qPCR

RNA was first extracted using Trizol reagent (Invitrogen), then followed by purification and DNase I digestion to remove genomic DNA contamination using RNAeasy column (Qiagen) according to the manufacturer's protocol. After reverse transcription (Thermo Scientific), cDNA were quantified with ABI 7900HT Fast Real-Time PCR System (Applied Biosystems). Relative gene expression levels were calculated using the ΔΔCt method, with 18S or GAPDH used as the reference gene and normalized. PCR Primer sequences will be available upon request.

#### Microarray method

Min6 cells were infected with Ad-EGFP or Ad-Ets1 for 48 hours. The cells were collected and total RNA was prepared by Trizol and RNA easy column (Qiagen). The RNA samples were sent to Shanghai Biotechnology Corporation for customer cDNA microarray analysis.

#### Chromatin Immunoprecipitation

Chromatin Immunoprecipitation (CHIP) was performed with a CHIP kit from Millipore according to the manufacturer's protocol using rabbit Ets1 antibody (Santa cruz) and positive RNA polymerase II antibody and negative control IgG provided in the kit. Min6 cells were fixed with 1% formaldehyde for 10 min, lysed in SDS lysis buffer and the chromatin was sonicated. PCR primers were designed around -400 bp upstream transcription start site of txnip gene promoter. PCR products were separated on agarose gel and imaged.

#### Luciferase assay

Luciferase assay was described before [28]. TXNIP promoter-luciferase reporter was co-transfected with CMV-Ets-1, pcDNA3-p300, pCMV-SPORT-βgal or empty vector pcDNA3.1. Results are shown as fold activation over reporter activity with empty vector. The chemiluminescent assay for β-galactosidase activity was performed by a Galacton-Star kit (Life Technologies). Results are shown as fold activation over reporter activity with empty vector.

#### EdU labeling and MTT assay

For EdU labeling, a 1∶1000 dilution of EdU labeling reagent (Invitrogen) was added to Min6 culture medium during the last 30 min of cell culture. EdU staining was conducted using Click-iT EdU imaging kit (Invitrogen) according to the manufacturer's protocol. BrdU labeling was used to show expression of Ets1 and BrdU labeling by immunofluorescence. BrdU was added to Min6 cells for 30 min and BrdU labeling was detected with mouse anti-BrdU monoclonal antibody (Roche). The MTT (3- [4,5-dimethylthiazol-2-yl]-2,5 diphenyl tetrazolium bromide) assay was performed by using a kit from Roche according to manufacturer's protocol.

#### Statistics

Statistical significance was assessed by two-tailed Student t test. A value of P<0.05 was considered significance. All data are expressed as the mean +/− SEM.

## Results

### Ets1 is enriched in β-cells in the mouse pancreatic islets

**Figure 1:**
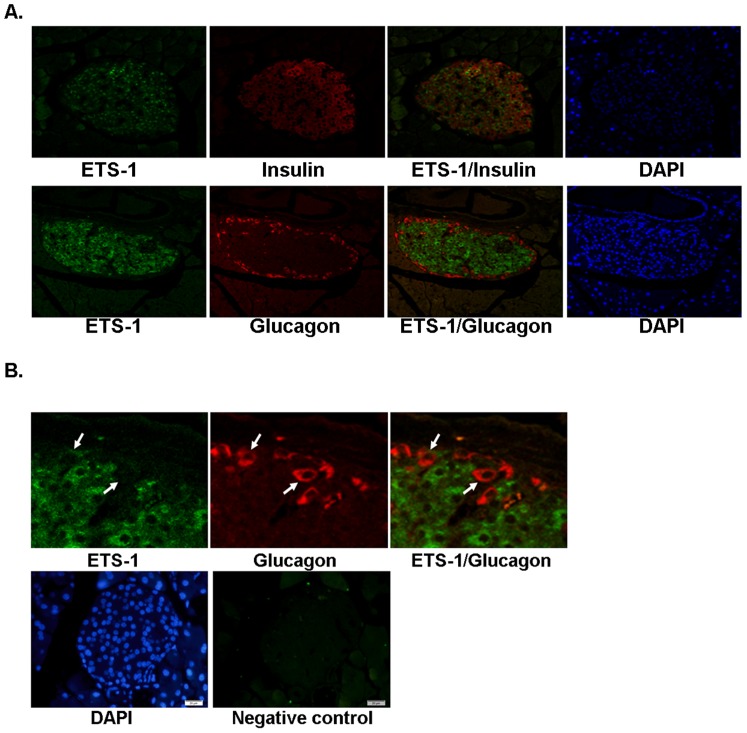
Ets1 is expressed in β-cells, but not in α-cells in mouse pancreas. (A). Paraffin-embedded pancreas sections from 16 weeks old mice fed chow diet were subjected to immunofluorescence analysis with rabbit anti-Ets1 antibody together with either mouse anti-insulin (top) or anti-glucagon (bottom) antibodies. Magnification 20X. (B). Higher magnification of section co-stained with Ets1 and glucagon antibodies. The arrows point at glucagon positive cells which did not express Ets1. The bottom panels showed cells stained with DAPI and anti-rabbit secondary antibody alone as a negative control. Magnification 40X.

To determine whether Ets1 is expressed in β-cells, we performed immunofluorescence in paraffin embedded mouse pancreatic tissue sections. Ets1 was found to be localized in insulin-positive, but not glucagon-positive cells (Fig. 1A and B). These results indicated that Ets1 was expressed in mouse pancreatic β-cells, but not in α-cells. Previous study show that serum response factor SRF is enriched in β-cells as well, suggesting that a general transcription factor can be restricted to β-cells within the islet and may play a role in regulation of β-cell function [30].

### Overexpression of Ets1 inhibits glucose-stimulated insulin secretion

**Figure 2:**
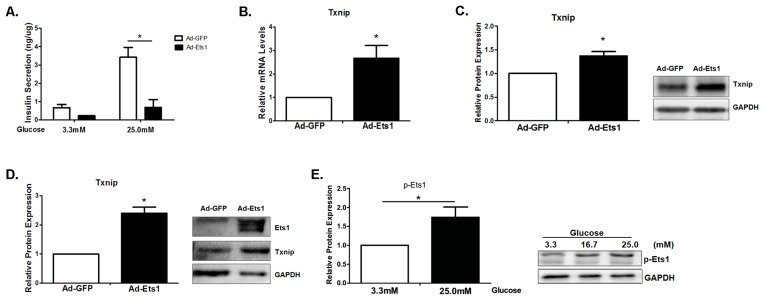
Ets1 inhibits glucose-induced insulin secretion and induces expression of TXNIP in mouse islets. (A). Adenoviral mediated overexpression of Ets1 inhibited glucose-stimulated insulin secretion in cultured mouse islet cells. Islets overexpressing EGFP were used as controls. Insulin concentration in the culture media was measured by mouse insulin ELISA kit as expressed as ng per µg of total islet protein. (n = 3/group; n is independent experiment). (B). TXNIP mRNA was induced by overexpression of Ets1 in Min6 cells as determined by RT-qPCR. (C). Overexpression of Ets1 induced TXNIP protein in Min6 cells. (D). Overexpression of Ets1 induced TXNIP protein in isolated mouse islets cultured under 20 mM concentration of glucose. (E). High glucose induced the phosphorylation of Ets1 in Min6 cells. Min6 cells were cultured under different glucose concentrations of 3.3, 16.7 and 25 mM. Endogenous Ets1 phosphorylation was detected by Western blot analysis with anti-Ets1-T38 phosphorylation antibody. GAPDH was used as loading control. (n = 3; values are shown as mean ± SD. *p<0.05, t-test)

Since Ets1 was mainly expressed in pancreatic β-cells, we hypothesized that it may play a role in impaired insulin secretion. To determine whether Ets1 inhibited glucose stimulated insulin secretion (GSIS), we overexpressed Ets1 in isolated mouse islets by adenovirus-mediated infection. Ets1 overexpression reduced insulin secretion both at low (3.3 mM) and high (25 mM) glucose concentration (Fig. 2A). Glucose at 25 mM stimulated insulin secretion at 3.5 ng/µg protein in control islets, but only at 0.8 ng/µg protein in Ets1 overexpressing islets; These results show that overexpression of Ets1 inhibited both basal and glucose stimulated insulin secretion dramatically.

### Overexpression of Ets1 stimulates TXNIP expression

To determine the target genes of Ets1 in β-cells, we infected Min6 cells with adenovirus expressing Ets1 (Ad-Ets1). cDNA microarray revealed that TXNIP was up-regulated by 5-fold in Min6 cells overexpressing Ets1. As TXNIP has been shown to play a role in GSIS, we surmised that Ets1 may inhibit GSIS through induction of TXINP. We confirmed the induction of TXNIP by 2.7-fold by overexpression of Ets1 in Min6 cells using RT-qPCR (Fig. 2B). Overexpression of Ets1 significantly increased TXNIP expression by ∼1.5-fold in Min6 cells (Fig. 2C) and by 2.5-fold in isolated mouse islets in culture (Fig. 2D). Overexpressed Ets1 was also detected by Western blot with Ets1 antibody (Fig. 2D). These results suggest that overexpression of Ets1 induced TXNIP expression in Min6 cells and mouse islets.

Since Ets1 is activated by ERK1/2-mediated phosphorylation, we wanted to determine whether high glucose concentration could induce phosphorylation of Ets1. Min6 cells were treated with increasing concentration of glucose at 3.3, 16.7 and 25 mM for 16 hrs and cell lysates were subjected to western blot analysis of Phospho-Ets1 T38. The result in Fig. 2E showed that 25 mM glucose induced ∼1.7-fold increase in the phosphorylation of Ets1 at T38. To exclude the possibility that this effect was due to increased osmolality, we adjusted equal osmolality with mannitol in cell culture medium and repeated the experiment. The result showed that under equal osmolality, high glucose concentration still induced phosphorylation of Ets1 (Fig. S1).

### Knockdown of Ets1 abolishes TXNIP expression in Min6 cells

**Figure 3:**
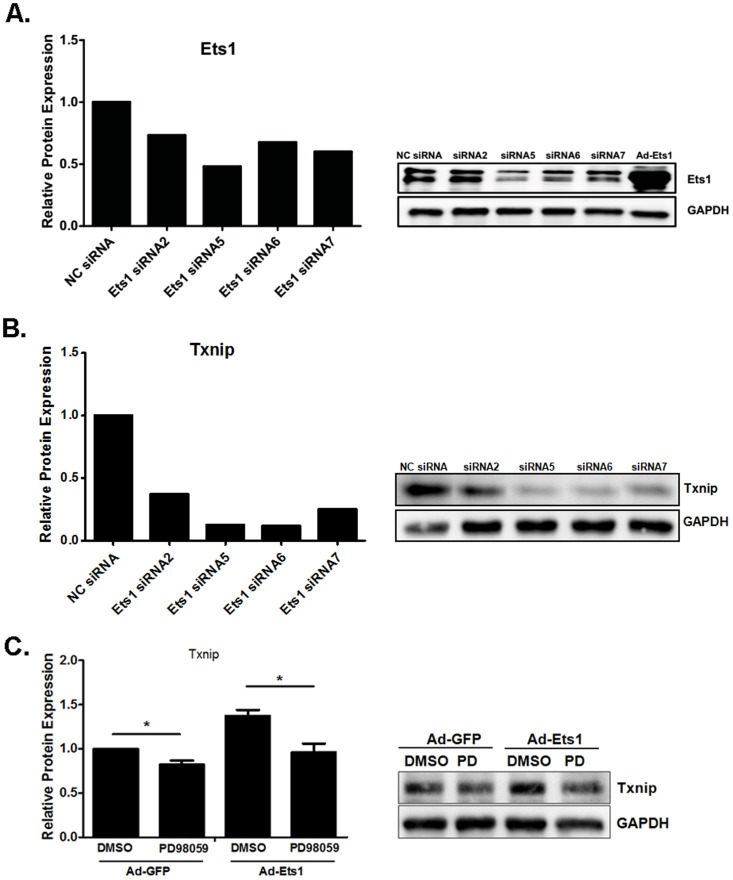
Endogenous Ets1 plays a role in TXNIP expression in Min6 cells. (A). Ets1 siRNA 2, 5, 6 and 7 silenced endogenous Ets1 protein in Min6 cells. Min6 cells were cultured in DMEM containing 25 mM of glucose. Scrambled siRNA and commercially designed siRNAs 2, 5, 6, 7 against mouse Ets1 were transfected into Min6 cells. After 48 hrs, the cell lysates were prepared and subjected to Western blot analysis with Ets1 antibody. (B). Knocking down of Ets1 resulted in reduced expression of TXNIP protein in Min6 cells as determined by Western blot. The cell lysates same as (A) were subjected to Western blot analysis with TXNIP antibody. (C). Inhibition of ERK1/2 by 20 µM of PD98059 blocked TXNIP expression in Min6 cells as determined by Western blot. n = 3.

To determine whether Ets1 is necessary for TXNIP expression in β-cells, we selected Min6 to perform siRNA treatment due to higher transfection efficiency in Min6 cells than that in isolated mouse islets. Min6 cells were transfected with Ets1 siRNAs under high glucose condition (25 mM). After 48 hours, we confirmed the knockdown efficiency by western blotting (Fig. 3A). Ets1 siRNA 5, 6, 7, but not the control siRNA or siRNA2, efficiently depleted endogenous Ets1 protein levels (Fig. 3A) and reduced TXNIP protein levels (Fig. 3B). We also use ERK1/2 inhibitor PD98059 to reduce phosphorylation and activation of Ets1. Pretreatment of Min6 cells overexpressing Ets1 with PD98059 blocked induction of TXNIP expression (Fig. 3C). These results indicate that endogenous Ets1 also regulated TXNIP expression in Min6 cells and that Ets1 phosphorylation by EKR1/2 was required for this effect.

### Ets1 associates with txnip gene promoter

**Figure 4:**
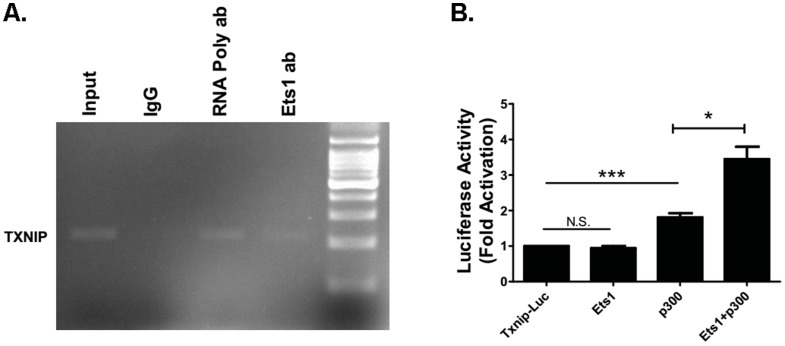
Ets1 is associated with txnip gene promoter and activates txnip promoter reporter with p300. (A). Endogenous Ets1 is associated with txnip promoter region (-400 bp) as shown by chromatin IP analysis in Min6 cells. RNA polymerase II antibody (shown as RNA Poly ab) was used as positive control and IgG was used as negative control. One percent of Input chromatin was also shown. (B). Ets1 and p300 activate txnip promoter luciferase reporter. HEK293 cells were transiently transfected with txnip proximal promoter (1 kb) luciferase reporter in the presence or absence of Ets1 and/or p300. (n = 3; values are shown as mean ± SD. *p<0.05, **p<0.01, ***p<0.001. N.S., not significant, t-test).

To determine whether Ets1 directly associates with txnip promoter in Min6 cells, we performed chromatin immunoprecipitation (CHIP) assay using Ets1 antibody. PCR primers were designed to amplify txnip promoter region that contains Ets1 binding sites at around -300 to -400 bp upstream transcription start site [25]. The CHIP results showed that endogenous Ets1 associated with the promoter region of Ets1 (Fig. 4A), suggesting that Ets1 may regulate TXNIP expression directly in Min6 cells.

### Ets1 and p300 synergistically activate txnip promoter reporter

It has been shown that p300 is necessary for Ets1 transcriptional activation [31]. To test whether Ets1 and p300 can synergistically activate the TXNIP promoter reporter, we transfected Ets1 expression construct with a 1 kb TXNIP proximal promoter reporter in HEK293 cells. Since HEK293 cells express high levels of endogenous Ets1 protein, overexpression of Ets1 alone did not activate the TXNIP promoter construct in transient transfection (Fig. 4B). On the other hand, overexpression of both Ets1 and p300 synergistically significantly activated TXNIP promoter reporter (Fig. 4B). These results suggest that Ets1 and p300 activated TXNIP promoter reporter expression in HEK293 cells.

### Overexpression of Ets1 inhibits proliferation of Min6 cells

**Figure 5:**
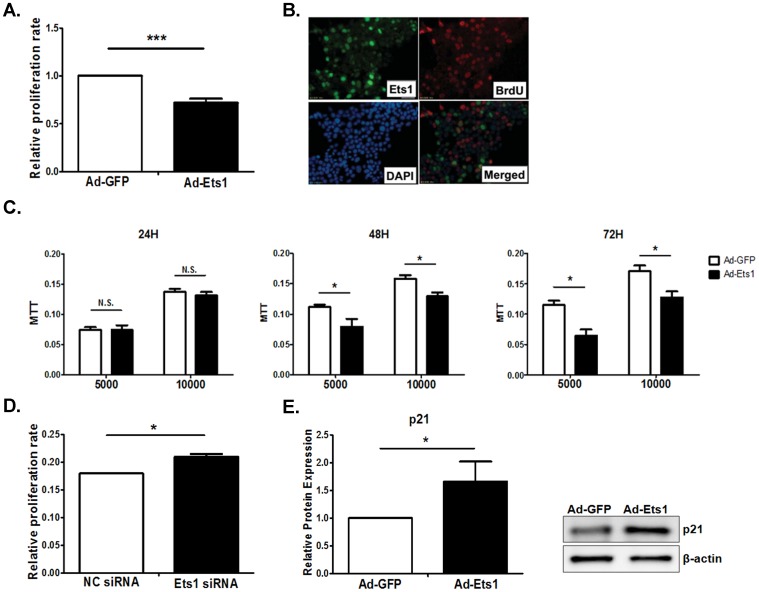
Ets1 inhibits Min6 cell proliferation. (A). Min6 cells were infected with Ad-EGFP or Ad-Ets1 for 24 hrs and cell proliferation was determined by EdU labeling. (B). Min6 cells were infected with Ad-Ets1 and incubated with BrdU. Overexpressed Ets1 was detected with rabbit anti-Ets1 antibody and BrdU labeling with mouse anti-BrdU antibody. (C). Min6 cell proliferation was also determined by MTT assay after 24, 48 or 72 hrs post-infection. (D). Min6 cells were transfected with Ets1 siRNA and cell proliferation was determined by EdU labeling. Ets1 siRNA treatment increased Min6 cell proliferation as shown by EdU labeling. (E). Ets1 induced p21 expression in Min6 cells. Min6 cells were infected with Ad-Ets1 and p21 protein levels were detected by Western blot. (n = 3; values are shown as mean ± SD. *p<0.05, **p<0.01, ***p<0.001. N.S., not significant, t-test).

Ets1 has been shown to stimulate cancer cell proliferation and inhibit endothelial cell proliferation through regulation of p21 [32]. Therefore we investigated the effect of Ets1 on Min6 cell proliferation. Overexpression of Ets1 reduced Min6 cell proliferation by ∼25% as detected by EdU labeling (Fig. 5A). In some experiments, we also used BrdU labeling to observe expression of Ets1 and BrdU labeling. Overexpressed Ets1 was detected by immunostaining with rabbit anti-Ets1 antibody, and BrdU labeling by mouse anti-BrdU antibody (Fig. 5B). Overexpression of Ets1 also reduced cell proliferation by ∼25% after 48 and 72 hrs of infection of Ets1 at two different cell density in the culture plate as detected by MTT assay (Fig. 5C). At 24 hr post-infection of Ad-Ets1, Ets1 did not inhibit cell proliferation, because Ets1 protein levels may not reach high levels. Conversely, silencing Ets1 by siRNA increased by ∼20% of Min6 cell proliferation as detected by Edu labeling experiment (Fig. 5E). Interestingly, the p21 cell cycle inhibitor was up-regulated by 1.5-fold in Min6 cells overexpressing Ets1 (Fig. 5E), suggesting a possible mechanism by which Ets1 inhibits Min6 cell proliferation.

## Discussion

In this study, we have identified Ets1 as a regulator of TXNIP expression in β-cells. Ets1 is expressed in β-cells and overexpression of Ets1 induced TXNIP expression whereas knockdown of Ets1 reduced TXNIP expression in Min6 cells. Ets1 was associated with txnip gene promoter and activated txnip promoter reporter in conjunction with p300. Therefore, we had identified Ets1 as a regulator that was involved in TXNIP expression and in the negative regulation of insulin secretion in β-cells.

TXNIP is a member of the alpha arrestin protein superfamily and serves as a links from ER stress to NIRP3 inflammasome activation in β-cells [12]–[14]. TXNIP has been shown to be positively regulated by CHREBP and negatively regulated by FOXO1 and Nuclear factor erythroid 2-related factor 2 (Nrf2) [33], [34]. FOXO1 blocks glucose-induced TXNIP expression and reduces glucose-induced ChREBP binding at the TXNIP promoter by competing with ChREBP for binding to the TXNIP promoter in β-cell. Interestingly, FoxO1 forms a complex with Ets1 in their DNA binding sites [35]. Nrf2 also represses both basal and glucose induced TXNIP expression by binding to txnip promoter and competing with MondoA in mouse heart [34]. TXNIP expression is suppressed during inflammatory responses [36]. TXNIP can also promote ChREBP nuclear translocation and induce its own expression in β-cells via positive feedback loop [37]. TXNIP induces islet amyloid polypeptide expression through MIR-124A and FOXA2 in β-cells [38].

It has been shown through second-generation sequencing that a number of histone acetyltransferases occupy TXNIP promoter [39], suggesting that MondoA-Mlx may recruit numerous ancillary factors to activate transcription [40]. Further, interaction between p300 and ChREBP has been demonstrated [16]. An ERK2 docking site is located on the Ets1 and phosphorylation of Ets1 enhances the binding affinity of Ets1 to CBP/p300 [41]. Ets1 contains both activation and repression domains, thus binding to CBP or p300 is critical for Ets1 to activate gene transcription [41]. Ets1 and p300/CBP synergistically activate Npr1 (encoding natriuretic peptide receptor-A) promoter [42], stromelysin promoter [22], Parathyroid hormone-related protein promoter [43]. Since p300 interacts with both Ets1 and CHREBP, it is also possible that Ets1 and CHREBP exist in a complex through p300 to activate TXNIP expression. Thus a multipartite complex of transcription factors and coactivators may regulate TXNIP transcription.

ERK1/2 mediated phosphorylation of Ets1 is important for the transcriptional activity of Ets1, and glucose induces ERK activity and phosphorylation of Ets1 (Fig. 2E), suggesting that glucose induction of TXNIP is at least partly through Ets1 transcriptional activation of TXNIP expression. In Min6 cells, previous study shows that knockdown of TXNIP by shRNA increases GSIS whereas overexpression of TXNIP inhibits GSIS [44]. TXNIP deficient mice exhibit increased GSIS in islets; in contrast, its overexpression suppresses glucose-induced adenosine triphosphate production, Ca(2+) influx and GSIS [45]. TXNIP enhances the expression level of UCP2 by recruitment of peroxisome proliferator-activated receptor-gamma co-activator-1alpha to the UCP2 promoter [18].

Recent study shows that Ets-1 activates expression of cyclooxygenase-2 in rat islet cells and overexpression of Ets1 by lentivirus inhibits insulin secretion [46], suggesting that ets1 could inhibit insulin secretion through multiple mechanisms.

TXNIP activates transcription of mir-204, which in turn targets and inhibits MafA expression, resulting in reduction of insulin gene expression in pancreatic β-cells [47]. MafA in turn controls insulin biosynthesis and secretion in β-cells and plays a role in glucotoxicity [3].

Taken together, the results of the present study suggest that Ets1 induces the expression of TXNIP and inhibits insulin secretion. These results suggest that Ets1 may be required for activation of NLRP3 and inflammasome in β-cells. In the future, it will be interesting to determine whether Ets1 regulates β-cells function in mice. Since TXNIP was found to be induced in β-cells in human type 2 diabetes [17], it will be interesting to determine whether phosphorylation of Ets1 is induced as well.

## Supporting Information

Figure S1 **Effect of high glucose concentration on phosphorylation of Ets1 T38 under equal osmolality condition in cell culture medium.** Min6 cells were treated with increasing concentrations of glucose and with mannitol to adjust equal osmolality in cell culture medium. The result showed that under equal osmolality condition, high glucose concentration increased Ets1-T38 phosphorylation. Anti-Phospho-Ets1 T38 antibody was used in Western blot. GAPDH was used as loading control.
(TIF)
