# Supplementary figures and images for: Transcription Factor Ets1 Regulates Expression of Thioredoxin-Interacting Protein and Inhibits Insulin Secretion in Pancreatic β-Cells
Source: PLoS One. 2014 Jun 4;9(6):e99049. doi: 10.1371/journal.pone.0099049 (PMC4045976; doi:10.1371/journal.pone.0099049)

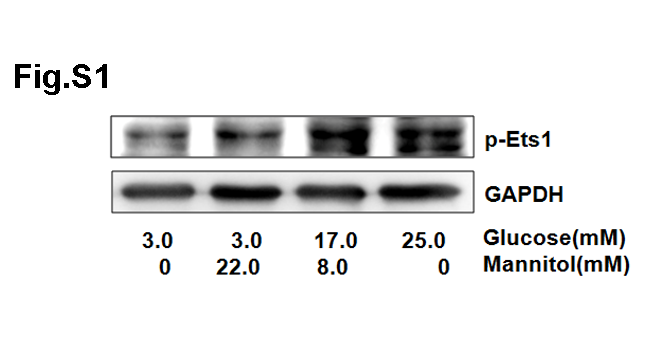

Supplement: Figure S1 — Effect of high glucose concentration on phosphorylation of Ets1 T38 under equal osmolality condition in cell culture medium. Min6 cells were treated with increasing concentrations of glucose and with mannitol to adjust equal osmolality in cell culture medium. The result showed that under equal osmolality condition, high glucose concentration increased Ets1-T38 phosphorylation. Anti-Phospho-Ets1 T38 antibody was used in Western blot. GAPDH was used as loading control. (TIF) [file pone.0099049.s001.tif]
